# Volatile Markers for Cancer in Exhaled Breath—Could They Be the Signature of the Gut Microbiota?

**DOI:** 10.3390/molecules28083488

**Published:** 2023-04-15

**Authors:** Manohar Prasad Bhandari, Inese Polaka, Reinis Vangravs, Linda Mezmale, Viktors Veliks, Arnis Kirshners, Pawel Mochalski, Emmanuel Dias-Neto, Marcis Leja

**Affiliations:** 1 Institute of Clinical and Preventive Medicine, University of Latvia, LV-1586 Riga, Latvia; reinis.vangravs@lu.lv (R.V.); linda.mezmale@lu.lv (L.M.); viktors.veliks@lu.lv (V.V.); arnis.kirsners@lu.lv (A.K.); marcis.leja@lu.lv (M.L.); 2 Riga East University Hospital, LV-1038 Riga, Latvia; 3 Faculty of Residency, Riga Stradins University, LV-1007 Riga, Latvia; 4 Institute of Chemistry, Jan Kochanowski University of Kielce, PL-25406 Kielce, Poland; pawel.mochalski@ujk.edu.pl; 5 Institute for Breath Research, University of Innsbruck, A-6850 Dornbirn, Austria; 6 Laboratory of Medical Genomics, A.C.Camargo Cancer Center, Sao Paulo 01508-010, Brazil; emmanuel@accamargo.org.br; 7 Digestive Diseases Center GASTRO, LV-1079 Riga, Latvia; 8 Faculty of Medicine, University of Latvia, LV-1586 Riga, Latvia

**Keywords:** breath analysis, volatile organic compounds, VOCs, breath biomarker, fecal microbiota, canonical correlation analysis, gastric cancer

## Abstract

It has been shown that the gut microbiota plays a central role in human health and disease. A wide range of volatile metabolites present in exhaled breath have been linked with gut microbiota and proposed as a non-invasive marker for monitoring pathological conditions. The aim of this study was to examine the possible correlation between volatile organic compounds (VOCs) in exhaled breath and the fecal microbiome by multivariate statistical analysis in gastric cancer patients (*n* = 16) and healthy controls (*n* = 33). Shotgun metagenomic sequencing was used to characterize the fecal microbiota. Breath-VOC profiles in the same participants were identified by an untargeted gas chromatography–mass spectrometry (GC–MS) technique. A multivariate statistical approach involving a canonical correlation analysis (CCA) and sparse principal component analysis identified the significant relationship between the breath VOCs and fecal microbiota. This relation was found to differ between gastric cancer patients and healthy controls. In 16 cancer cases, 14 distinct metabolites identified from the breath belonging to hydrocarbons, alcohols, aromatics, ketones, ethers, and organosulfur compounds were highly correlated with 33 fecal bacterial taxa (correlation of 0.891, *p*-value 0.045), whereas in 33 healthy controls, 7 volatile metabolites belonging to alcohols, aldehydes, esters, phenols, and benzamide derivatives correlated with 17 bacterial taxa (correlation of 0.871, *p*-value 0.0007). This study suggested that the correlation between fecal microbiota and breath VOCs was effective in identifying exhaled volatile metabolites and the functional effects of microbiome, thus helping to understand cancer-related changes and improving the survival and life expectancy in gastric cancer patients.

## 1. Introduction

The gut microbiota plays an important role in maintaining the healthy metabolism in the host. This regulation of the host health relies on the production of different classes of metabolites by the microbiota and their underlying molecular mechanisms in both healthy and diseased conditions [1,2]. Therefore, microbiota-derived metabolites are direct mediators of the microbe–microbe interactions and host–microbial interactions. The presence and abundance of these diverse metabolites that can affect our health are subject to the microbiota composition, in addition to dietary intake, drug use, and environmental factors [3]. The volatile organic compounds (VOCs) released within the gut as metabolites are absorbed into the bloodstream, distributed amongst tissues and organs, transported to the lungs, and subsequently, excreted via the exhaled breath. This suggests that breath VOCs could provide a valuable insight into the gut microbiota and disease diagnosis [4].

The gut microbiome has been linked with a plethora of diseases, gastrointestinal disorders, and different types of cancer, including gastric cancer and colorectal cancer [5,6,7]. This may lead to the deviation of various metabolic pathways and altered concentrations of certain VOCs reflected in different bodily fluids, cell lines, and tissues, including exhaled breath.

The identification of volatile metabolites as potential biomarkers of a disease through non-invasive analysis still remains a challenge. Breath analysis is an easy, non-invasive, real-time, and innovative method used to analyze the chemical composition of exhaled breath [8,9]. In particular, VOCs are helpful in disease diagnosis and have great potential as indicators of metabolic disorders, disease onset, and progression [10,11,12,13]. Furthermore, breath VOCs provide a source of useful biomarkers with possible associations to the body’s metabolism, along with the fecal microbes that contribute to numerous molecular mechanisms [14]. Therefore, the analysis of volatile metabolites in the breath could be used as a diagnostic and monitoring tool for diverse gastrointestinal diseases and cancers.

Several well-established volatile breath metabolites, such as aldehydes, ketones, and hydrocarbons; bacterial fermentation products, including alcohols; and short-chain fatty acids (SCFAs), such as acetate, butyrate, and propionate have been correlated with gut microbiota and are exhaled via the breath [15,16]. However, the origins of volatile metabolites and the intermediate products are not fully understood. The VOCs emitted by feces in the gastrointestinal tract could be transported into the blood and appear, finally, in the exhaled breath [17]. The human gut microbiome is composed of 10–100 trillion microorganisms with more than 400 bacterial species belonging to predominant phyla, such as *Actinobacteria*, *Bacteroidetes*, *Firmicutes*, *Proteobacteria*, *Verrucomicrobia*, *Fusobacteria*, and *Synergistetes* [18]. Gut microbes are exogenous sources of ethanol, which can be detected in the breath. An impaired gut microbiome and the implicated bacteria, such as *Fusobacterium nucleatum*, *Escherichia coli*, and *Bacteroides fragilis* were correlated with colorectal cancer [19]. Several facultative anaerobic species, such as *Dialister invisus*, *Parabacteroides* spp., Lachnospiraceae, and Ruminococcaceae, as well as strains such as *Faecalibacterium prausnitzii* and *Clostridium clostridiiforme,* have been associated with metabolite disturbances, including SCFAs, bile acids (BAs), choline, and specific pathways in several gastrointestinal and inflammatory disorders [20,21,22]. The gut microbiota is likely to play a significant role in the advancement and progression of gastric cancer.

In the last decade, the advancement and the application of metagenomics, metabolomics, breathomics, and omics data analysis (i.e., multi-omics approaches) have led to a better understanding of the fecal and breath volatilome and their contribution to pathogen virulence and overall gut health [23]. A number of efforts have been made to identify breath markers for pathogenic bacteria infecting the gut [24,25,26]. However, the main challenges in this context are the non-specificity of these VOCs and the confounding factors that prevent the use of these volatile markers in disease diagnostics. Understanding the complex and underlying biochemical mechanisms that link the fecal microbes to exhaled VOCs could help to illustrate the disease pathology and develop novel therapies and screening methods.

Studies have identified over 2700 VOCs in human breath [27]. Surprisingly, to date, a breath signature that reflects the interaction between fecal microbiota and breath metabolites within the context of gastric cancer has not been studied. In this article, we present an integrated statistical approach used to investigate possible correlations between breath VOCs, as identified by gas chromatography–mass spectrometry (GC–MS), and fecal microbiota, through sequencing, in gastric cancer patients and healthy controls. In general, some of the specific breath metabolites were likely to have a direct or indirect link with the fecal microbiota composition and their presence or abundance varied between gastric cancer patients and healthy controls.

## 2. Results

### 2.1. Study Population

The analysis included a total of 49 participants. All participants provided both breath and fecal samples. Out of these participants, 16 were gastric cancer patients and 33 were healthy controls. The patient group showed different clinical stages of gastric cancer, starting at IA and going up to IV. An overview of clinical and demographic data is presented in Table 1. The data are presented as percentages or medians ±.

### 2.2. Microbiome Analysis

**Figure 1:**
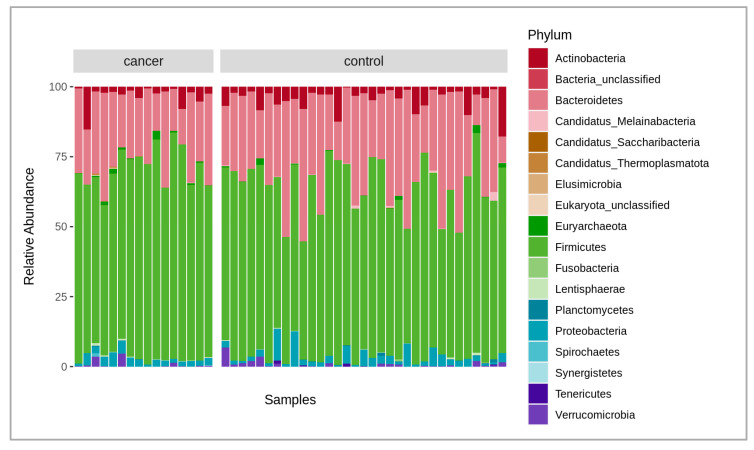
Stacked bar plot of relative abundances of different dominant phyla in both cancer and control groups, where the *x*-axis represents the samples, and the *y*-axis, the relative abundance of the phyla.

A total of 6.2 billion raw reads were obtained from all the samples analyzed. After quality filtering, trimming (mean quality score of 30 and above 50 bp read length), and host sequence removal, 6 billion paired reads remained with an average of 123 million paired reads per sample, in a range between 86 and 178 million. The negative control added during the extraction did not provide enough DNA for library construction, thus indicating a low possibility of reagent and human contamination. Overall, there were 1324 bacterial species identified after the initial analysis of the microbiome composition using the MetaPhlAn4 marker database. At the phylum level, the fecal microbiota of cancer and the control patient samples were, on average, dominated by *Bacteroidetes* at 24.9% and 30.2%; *Firmicutes* at 67.5% and 60.7%; *Actinobacteria* at 3.3% and 3.5%; and *Proteobacteria* at 2.6% and 3%, respectively (Figure 1). After obtaining data from both Bracken and MetaPhlAn4, we compared the results and found them to be highly similar. As a result, we decided to perform further taxonomic analysis using the MetaPhlAn4 data exclusively. A permutational multivariate analysis of variance (PERMANOVA) was conducted to determine if the taxonomic data obtained from MetaPhlAn4 or Bracken revealed any significant differences between the fecal microbiomes in cancer patients and the control group. The results revealed that neither the MetaPhlAn4 nor the Bracken-obtained taxonomic data showed that the patient group (control or cancer) was the main driver of the observed microbiome variance among individual samples (*p*-value < 0.05), as in both cases only 4.7% and 3.0% of the variance could be explained, respectively.

Figure 1 shows the comparison of the fecal microbiota between the gastric cancer patients and the healthy controls. The relative abundance of the compositional differences in the bacteria phyla in the cancer and control groups are depicted in the figure.

In order to remove possible false-positive results (misidentifications) and rare species that have little importance for the downstream breath VOC analysis, only the species present in at least 30% of the samples were retained for the analysis, which left 395 bacterial species in the analysis dataset. The taxonomic analysis was performed on the full dataset.

**Figure 2:**
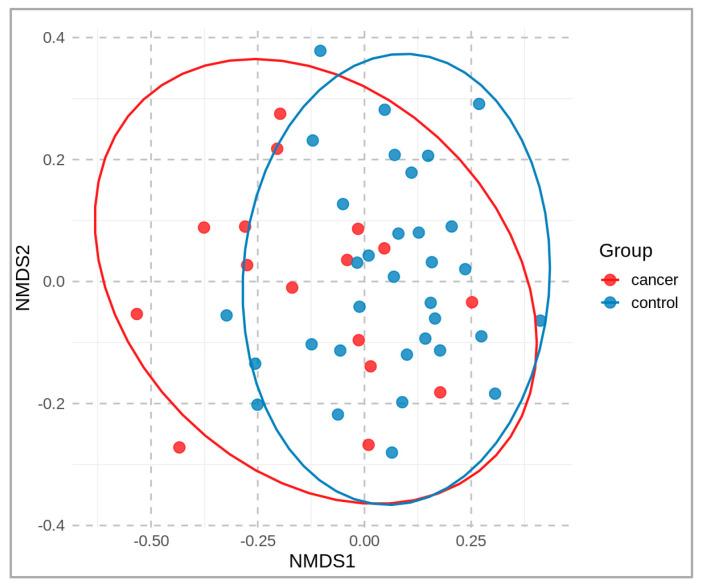
Two-dimensional NMDS plot of fecal microbial communities, based on beta-diversity (OTU level), derived from fecal samples of cancer patients (red) and healthy controls (blue).

A non-metric multidimensional scaling (NMDS) plot, colored according to the sample groups, is presented in Figure 2. It compares the microbial community of cancer patients vs. healthy controls at the operational-taxonomic-unit (OTU) level. A distance matrix of the sample data was calculated using the Bray–Curtis dissimilarity index, and the NMDS was performed using two dimensions (stress 0.19). The resulting two-dimensional plot (NMDS1 and NMDS2) is shown, with each point representing a sample that is colored according to its group.

**Figure 3:**
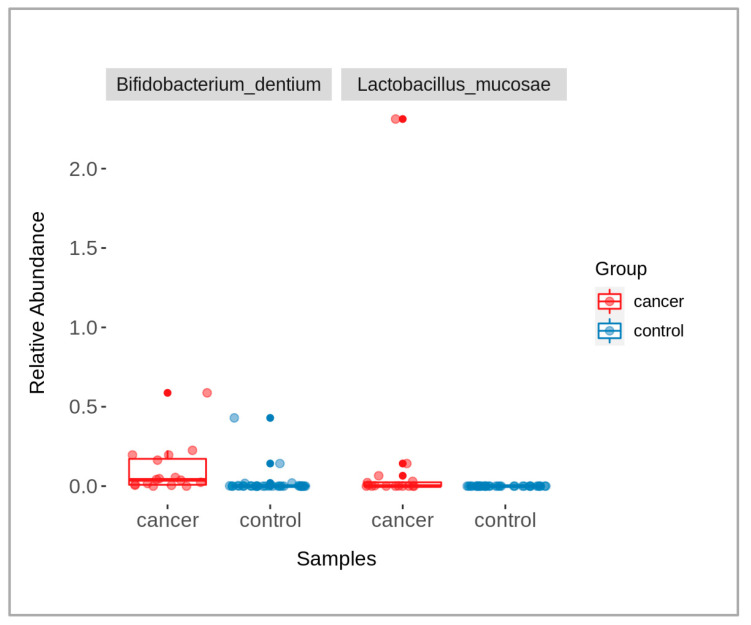
A box-plot showing the presence of significantly different bacterial taxa between cancer (red) and control (blue) groups (*p*-value < 0.05).

When comparing data of the patients and controls, some microbial taxa were found with significantly abundant differences (*p*-value < 0.05), namely *Bifidobacterium dentium* and *Lactobacillus mucosae* (Figure 3). The taxa were identified using a Wilcoxon rank-sum test, followed by a Benjamini–Hochberg correction for multiple testing. The graph shows the relative abundance of each of these taxa across the two groups, with box-plots indicating the median and interquartile ranges and individual points representing the samples.

### 2.3. Analysis of Breath VOCs

Altogether, 378 VOCs were detected in the exhaled breaths of the participants using GC–MS. Since the majority exhibited low occurrence, only VOCs that were present in at least 20% of the samples were used for further analysis. Effectively, there were 116 breath volatile metabolites in the analysis dataset.

After a sensitivity analysis by backwards elimination of the least-contributing volatile metabolites, the most important metabolites identified in this study were selected, resulting in sets containing 14 and 7 breath VOCs for the cancer and control groups, respectively. These VOCs contributed the most to the overall correlation between the two datasets and were designated as putatively relevant in the interaction with fecal microbiota. Interestingly, none of these reported metabolites were common across the two groups.

**Figure 4:**
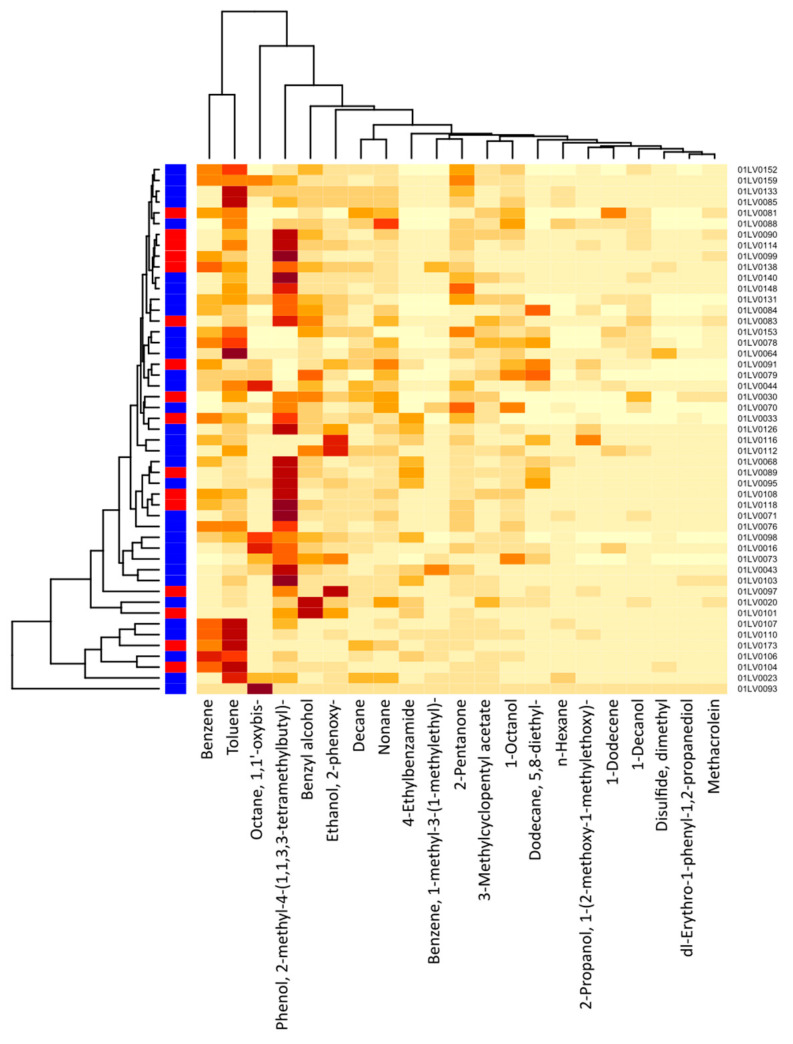
Cluster heatmap of breath VOC levels for each study participant (21 VOCs and 49 participants). The *x*-axis depicts the respective breath VOCs and the *y*-axis, the patient IDs. Red color on the left vertical axis indicates gastric cancer cases, whereas blue color indicates healthy controls. The darker-colored cells indicate higher concentrations of metabolites, and lighter-colored cells represent lower concentration levels for each subject. The intensity of color corresponds to the concentration levels of VOCs.

Figure 4 shows a cluster heatmap of the breath volatile metabolites identified in the breath samples of all 49 participants. The VOCs identified in the exhaled breath of the gastric cancer patients were the following: 1-dodecene; 1-octanol; 2-pentanone; 2-propanol, 1-(2-methoxy-1-methylethoxy)-; benzene; 1-methyl-3-(1-methylethyl)-benzene; n-decane; dimethyl disulfide (DMDS); 5,8-diethyl-dodecane; 2-phenoxy-ethanol; n-nonane; 1,1′-oxybis-octane; toluene; and n-hexane. Similarly, the VOCs identified in the exhaled breath of the healthy controls were the following: 1-decanol; 3-methylcyclopentyl acetate; 4-ethylbenzamide; benzyl alcohol; methacrolein; phenol, 2-methyl-4-(1,1,3,3-tetramethylbutyl)-; and dl-erythro-1-phenyl-1,2-propanediol. The major compounds showing higher concentration levels and observed in most of the subjects were phenol, 2-methyl-4-(1,1,3,3-tetramethylbutyl)-; toluene; benzyl alcohol; benzene; 1,1′-oxybis-octane; 2-phenoxy-ethanol; 2-pentanone; and n-nonane.

**Figure 5:**
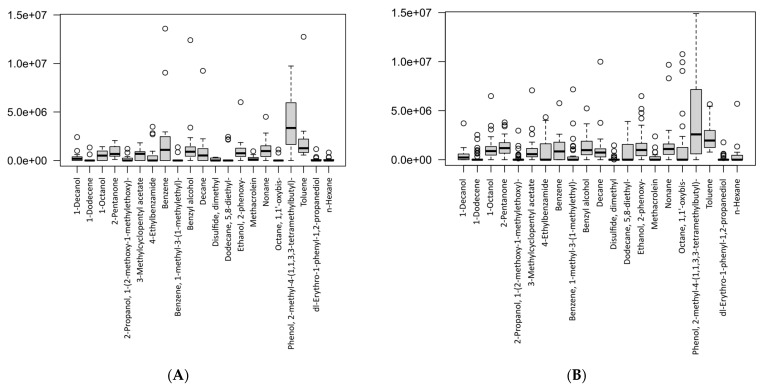
Box-plots showing the VOC concentrations according to study groups: (**A**) gastric cancer patients, (**B**) healthy controls. Data are expressed as medians and interquartile range (IQR), minimum–maximum, and extreme values or outliers (*p*-value < 0.05 is considered as statistically significant).

The differences in the VOC concentrations between the two study groups are presented in the box-plots in Figure 5. Figure 5A shows the levels of breath metabolites found in the gastric cancer patients, whereas Figure 5B shows the concentration levels of the breath metabolites in the healthy controls. Therefore, the plots showed the levels of breath VOCs that had been selected after the canonical correlation analysis (CCA) in the cancer and control groups, separately. Furthermore, this suggested that the VOCs were significant, as determined by the sensitivity analysis on each group.

A table with the descriptive statistics and *p*-values for the respective VOCs between the groups is presented in Table 2. We found that for most of the VOCs, there were no significant differences. However, two volatile metabolites were significantly different between the cancer and control groups, i.e., 1-octanol (*p*-value = 0.033) and octane, 1,1′-oxybis- (*p*-value = 0.027). We also identified that these two metabolites did not overlap between the study groups, even after a sensitivity analysis, and were present exclusively in cancer cases. These results demonstrated that 1-octanol and octane, 1,1′-oxybis- could be potential breath biomarkers for gastric cancer. Importantly, studies had previously associated these VOCs in exhaled breath with a number of bacteria-related metabolic pathways associated with cancer [4,28,29,30].

### 2.4. Canonical Correlation Analysis of VOCs and Microbiome Data

To reduce the dimensionality of the dataset and obtain a more similar number of features and samples, the feature space of both the breath-VOC and microbiome data subsets were transformed using a sparse principal component analysis (S-PCA).

**Figure 6:**
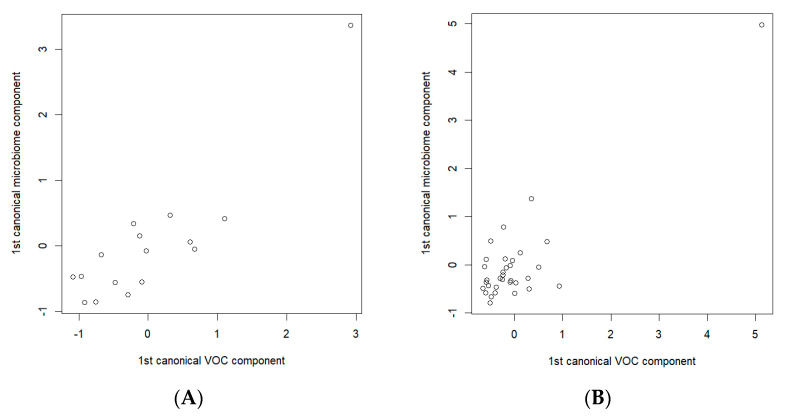
Canonical correlation score plot between the selected breath VOCs (the *x*-axis) and fecal microbiome composition (the *y*-axis) using the first canonical components for (**A**) cancer patients; (**B**) healthy controls. CCAs were performed on the sets of 14 and 7 breath VOCs, and 33 and 17 bacterial species, of the cancer patients and healthy controls, respectively.

To detect the possible relationships between the breath VOCs and the fecal microbiota specific to gastric cancer, separate analyses of the data related to the cancer patients and the data of the control group were carried out. The CCA analysis of the S-PCA transformed data showed a statistically significant correlation between the components (a correlation of 0.891, *p*-value = 0.045, in the cancer data subset and a correlation of 0.871, *p*-value = 0.0007, in the control data subset). Figure 6 depicts the resulting CCA score plots of the cancer patients (Figure 6A) and healthy controls (Figure 6B). In the figures, the *x*-axis represents the canonical variate for the breath VOCs, whereas the *y*-axis represents the canonical variate for the fecal microbiome data. Each point corresponds to integrated data from the exhaled breath and the fecal samples collected from the same participants.

After a sensitivity analysis by backwards elimination of least-contributing VOCs and bacteria (i.e., the removal of VOCs/bacteria that did not have a significant impact on the correlation and its statistical significance), there were 14 VOCs and 33 species of bacteria remaining in the cancer subset and 7 VOCs and 17 species of bacteria in the control subset. Similarly, we applied a threshold of ≥0.5 for the correlations in both datasets to obtain the final and most important exhaled VOCs and fecal bacterial taxa.

The analyses of the VOCs and fecal bacteria were continued by analyzing the significant correlations ≥ 0.5 and by investigating their biological relevance in the available literature, as well as in the metagenome sequencing results. The VOCs were evaluated according to the pathways of the bacteria with which they were correlated in order to determine whether they could explain the observations related to the specific VOCs and their potential roles in cancer.

The chemical identities of the 21 exhaled volatile metabolites that had the highest contribution to the overall correlation, found in both the cancer and control groups, are shown in Table 2. We found that none of the volatile metabolites were common between cancer and control groups, whereas only one fecal bacterial species (i.e., *Clostridium_SGB6179*) overlapped between the two groups.

**Figure 7:**
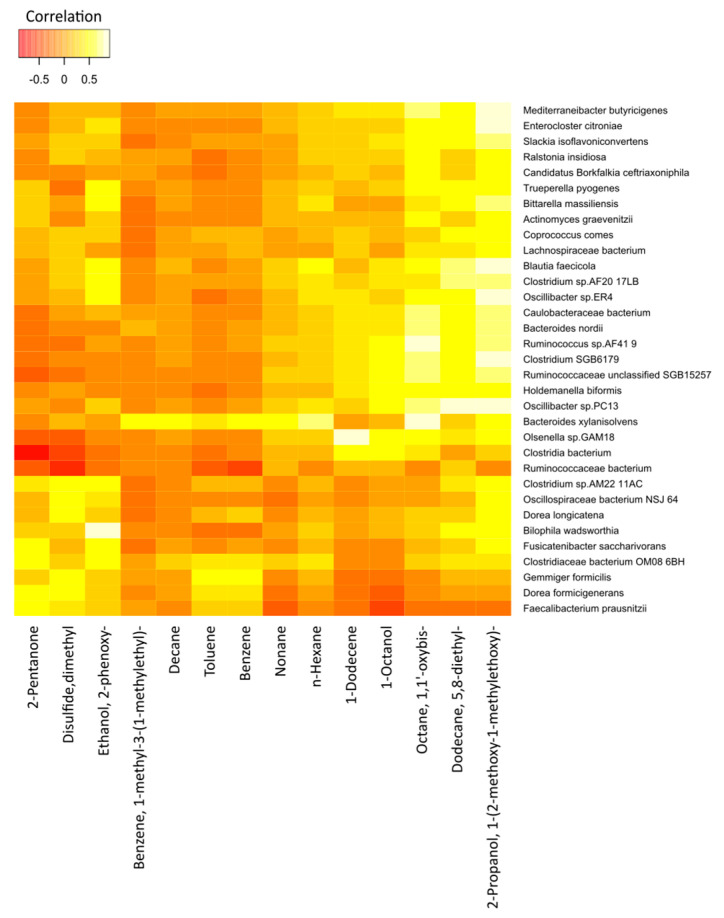
Correlation heatmap of the 14 VOCs and 33 fecal bacterial species in cancer patients. Red color indicates negative correlations, and white or light yellow color indicates positive correlations. The quantitative color scheme is shown at the top.

**Figure 8:**
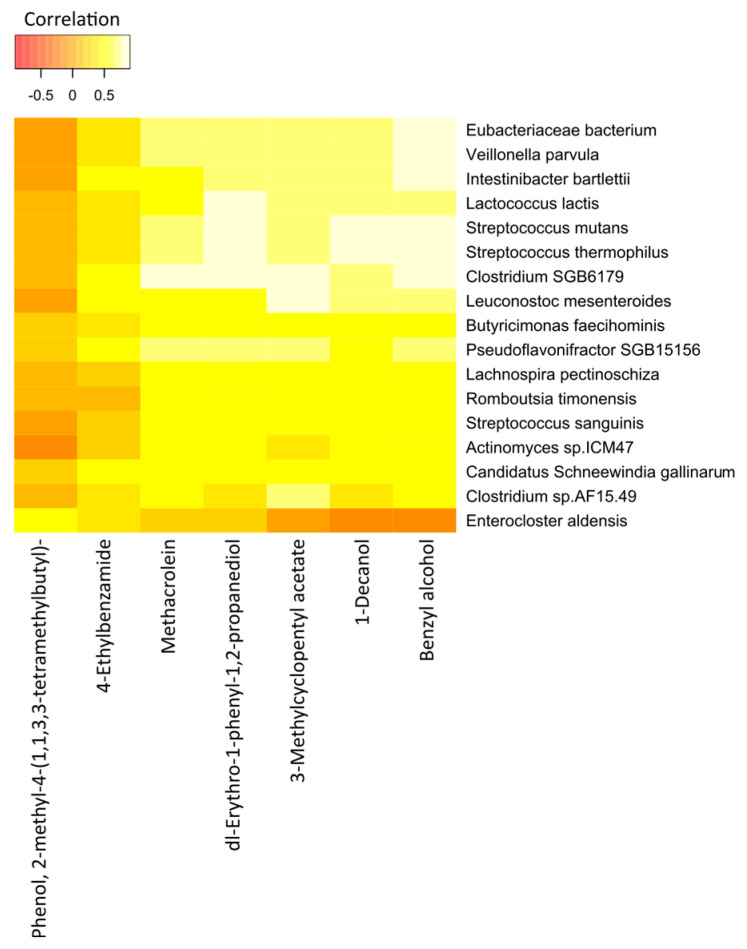
Correlation heatmap of the 7 VOCs and 17 fecal bacterial species in healthy controls. Red color indicates negative correlations, and white or light yellow color indicates positive correlations. The quantitative color scheme is shown at the top.

The correlations between the most significant volatile components and the detected species of bacteria in the two study groups are represented in Figure 7 (cancer cases) and Figure 8 (controls), by correlation heat maps. Interestingly, in the cancer subset, we observed a higher number of bacterial species and exhaled volatile metabolites, as compared to those in the control subset. This outcome possibly indicated distinct microbiome compositions and subsequent breath metabolites across both disease and healthy states.

In Figure 7, an exhaled-breath-matrix dataset based on the fecal bacteria taxa in gastric cancer patients is shown. Based on the correlation heatmap, it was apparent that a vast majority of the individual correlations between the breath VOCs and the fecal bacteria were positive, which was indicated by the trend of lighter yellow, as compared to the other VOC columns. Of the 14 preselected breath VOCs in the 16 cancer patients, we observed that the VOCs, such as 2-propanol, 1-(2-methoxy-1-methylethoxy)-; 5,8-diethyl-dodecane; octane, 1,1′-oxybis-; 1-octanol; 1-dodecene; n-hexane; and n-nonane, in particular, were highly correlated with the abundance of microorganisms. Similarly, the levels of benzene; toluene; n-decane; and benzene, 1-methyl-3-(1-methylethyl)- in the breath correlated with the abundance of the *Bacteroides xylanisolvens* and *Gemmiger formicilis* bacteria. In addition, 2-pentanone, a ketone, was clearly linked to the *Dorea formicigenerans* and *Clostridiaceae bacterium_OM08_6BH* strains. We observed dimethyl disulfide correlated to the *Dorea longicatena*, *G. formicilis*, *D. formicigenerans*, *Clostridium_sp_AM22_11AC*, and *Oscillospiraceae bacterium_NSJ_64* species. Furthermore, ethanol, 2-phenoxy- in the exhaled breath of cancer patients was related to *Fusicatenibacter saccharivorans*, *Oscillibacter_sp_ER4*, *Clostridium_sp_AF20_17LB*, *Bilophila wadsworthia*, *Bittarella massiliensis*, *Blautia faecicola*, and *Clostridium_sp_AM22_11AC*.

Furthermore, after comparing the rows, different bacterial species were significantly different from each other. Bacteria species, such as *B. xylanisolvens*, *Ruminococcaceae unclassified_SGB15257*, *Holdemanella biformis*, and *Olsenella_sp_GAM18,* trended towards the white color or had strong positive correlations with a majority of the breath volatile metabolites, as compared to the other bacteria.

The correlation heatmap for the breath VOCs and the fecal bacterial taxa that contributed most to the correlations in the control group is shown in Figure 8.

Interestingly, we found statistically significant correlations between the individual breath VOCs and fecal bacteria. Of the 7 preselected VOCs from the 33 healthy controls, benzyl alcohol; dl-erythro-1-phenyl-1,2-propanediol; 1-decanol; methacrolein; and 3-methylcyclopentyl acetate were strongly correlated with the abundance of most of the bacterial species under observation. Likewise, except for *Enterocloster aldensis* and *Actinomyces sp_ICM47*, all other bacterial species exhibited strong correlations with the levels of VOCs previously mentioned.

The correlation tables showing the magnitudes of the correlation coefficients between the breath metabolites and fecal bacterial taxa in the cancer and control subsets are provided in Tables S1 and S2, respectively, in the Supplementary Materials.

## 3. Discussion

The present study aimed to investigate the possible correlations between specific breath VOCs, as identified by GC–MS, and the gut microbiota, as evaluated by shotgun metagenomics sequencing, in relation to gastric cancer. The exhaled breath samples and the fecal samples from a total of 49 participants (16 cancer, 33 controls) were analyzed. We hypothesized that the breath VOCs would be related to the fecal microbiota composition, directly or indirectly, and their presence and levels would vary between the disease and healthy states.

Altogether, our results revealed a significant relation between the fecal microbiota and the volatile metabolites in exhaled breath, as they differed between the cancer patients and the healthy controls. The exhaled breath analysis was integrated with the fecal microbiome analysis, and together, they could serve as non-invasive diagnostic-and-screening tools for gastric cancer, as this disease has been associated with changes in the volatile metabolic profiles and dysregulation of the gut microbiota, its composition, and the related metabolic pathways [31,32]. However, the mechanisms underlying the presence of a specific breath VOCs detected in cancer patients, along with links to gut microbiota activities, have not yet been elucidated. Several studies have suggested hypotheses regarding the generation of various VOCs. Typically, these are produced by the gut microbes via enzymatic reactions, fermentation, and normal metabolism, giving rise to SCFAs, ketones, alcohols, phenols, branched-chain fatty acids (BCFAs), sulfides, bile acids, branched hydrocarbons, and aldehydes, amongst others [33,34,35].

While microorganisms are present throughout the human digestive tract, studying their presence in fecal samples is crucial to understand the composition of fecal microbiota and their interactions with the host and their contributions to host phenotypes [36]. Although numerous studies have analyzed VOCs in different biological matrices for disease detection [37,38,39], the influence of fecal microbiota on human breath profiles and their complex interactions had not been previously investigated within the context of gastric cancer detection. In the current study, we analyzed the correlations between biologically relevant breath VOCs and gastric cancer-associated fecal microbiota for the first time in patients with gastric cancer, as compared to healthy controls. Interestingly, we discovered significant correlations between the profiles of breath VOCs and fecal microbes in the cancer and control subjects under study. It was evident that a majority of the exhaled breath VOCs could have been related to the fecal microbiota composition and abundance, for example, through the microbial metabolism of the host metabolites. Furthermore, the volatile metabolites in the exhaled air reflected the state of the gut homeostasis and the environmental alterations to the structure of the fecal microbiome community [40].

Recent studies have shown that the concentrations of VOCs in the breath and the abundance of fecal microbes differed according to the state of gastrointestinal diseases. Smolinska et al. highlighted a strong link between the specific VOCs in exhaled breath and the specific fecal bacterial strains in a study that included a cohort of patients with Crohn’s disease, a type of inflammatory bowel disease [41]. The same authors found that there was a significant difference in the breath VOCs and the diversity of the fecal microbiota between the active and inactive stages of Crohn’s disease [42] and ulcerative colitis [43]. This indicated that the relation was dependent on the disease stage. Profiling VOCs in exhaled breath to identify potential biomarkers for gastrointestinal diseases has been the focus of numerous studies. Previous research has associated the levels of different VOCs with the changes and the abundances of different fecal microbes [44]. Despite the large variations in the outcomes among studies, breath analyses have provided the most patient-friendly platform and achieved the highest participation rates [31].

In this study, we found distinct breath metabolites and microbiota compositions, as well as distinct correlations, in the analyzed samples of the cancer and control groups. This suggested different underlying metabolic processes or mechanisms could exist between the groups. The CCA analysis showed a slightly stronger correlation in cancer cases (correlation 0.891) than in the healthy controls (correlation 0.871). Similarly, a larger number of breath VOCs and bacterial species were observed in the cancer patients than in the controls, once again attesting to the distinct microbiome compositions and metabolisms associated with each group. Interestingly, the observed VOCs did not overlap between the groups, whereas a single bacterial taxon was present in both; therefore, the correlations found in this study were dependent on the state of disease or health. Long-term dietary patterns could alter microbiota composition and metabolic activity [45]. Herein, we found several ketones, branched hydrocarbons, alkanes, benzenes, sulfides, phenols (protein fermentation), alcohols, unsaturated aldehyde (methacrolein), aromatics, and bacterial fermentation products in the exhaled breath, contributing to the overall correlations with the fecal microbiota. All have been reported to be associated with several gastrointestinal disorders and cancers [46,47]. However, a shared presence among the related individual VOCs in gastric cancer was not found.

Similar to other studies of gastrointestinal disorders, we found statistically significant correlations among specific hydrocarbons (n-decane, n-nonane, n-hexane, 5,8-diethyl-dodecane), one ether (octane, 1,1′-oxybis), and bacteria *B. xylanisolvens* and *H. biformis,* in cancer patients. *B. xylanisolvens* has been linked with high-fiber dietary patterns [48]. The metabolic pathways responsible for the formation of these hydrocarbons remain unclear. Nevertheless, they might emanate from the lipid peroxidation processes or from environmental exposure, and they have been associated with *Helicobacter pylori* and *Mycobacterium avium*. Furthermore, 2-pentanone, commonly reported in the breath and fecal samples in numerous studies, was produced via the β-oxidation of fatty acids or the oxidation of 2-pentanol catalyzed by either alcohol dehydrogenases (ADHs) or cytochrome p450 (CYP2E1) [49,50]. The source of this compound may be the bacterial action in the gut. In our study, anaerobes *D. formicigenerans* and *Clostridiaceae bacterium_OM08_6BH* of *Firmicutes* revealed significant correlations with 2-pentanone. The *D. formicigenerans* strain has been associated with obesity-related inflammation.

Prebiotic supplementation with pectin, a dietary fiber, did not have an impact on fecal microbiota composition, SCFAs, or exhaled VOCs in young adults versus elderly subjects [51]. Low-fiber, fat, and high-protein diets were related to an increase in proteolytic fermentation, resulting in shifts in the gut microbiota [52]. In turn, detrimental amino acids and proteolytic fermentation products (e.g., BCFAs) increased in concentration, while beneficial SCFAs were lowered. Host glycans and mucins can be degraded by adverse gut microbes in the intestinal lumen, which maintains the gut barrier integrity, the impairment of which may give rise to enhanced terpenes, aldehydes, and ketones. Furthermore, this caused low-grade systematic oxidative stress, and the microorganisms generated metabolites related to the tricarboxylic acid (TCA) cycle and the Warburg metabolism [31,53].

DMDS, a sulfur-containing compound, was most likely related to the proteolytic fermentation by human fecal microbiota and is an important breath biomarker [54]. Indeed, enhanced levels of sulfides were observed in fecal microbiome and VOC-related studies. DMDS could be synthesized by different microbes in the intestinal tract via common biochemical pathways [55]. The dominant species in the human gut, such as *D. longicatena*, *D. formicigenerans*, *G. formicilis*, and *Clostridium_sp,* showed a strong positive correlation to DMDS. However, the relation of DMDS to *G. formicilis* had not been demonstrated previously. Complex benzenes and toluene, as volatile aromatic hydrocarbons, were reported in higher concentrations in human breath, urine, and blood, and have been commonly attributed to environmental exposure, smoking, and pathogens, such as *H. pylori*, *Pseudomonas aeruginosa*, and *M. avium* [56,57]. In our study, 1-methyl-3-(1-methylethyl)-benzene was significantly correlated to *B. xylanisolvens*, whereas benzene and toluene were sparsely correlated to the *G. formicilis* species. *B. xylanisolvens* was shown to degrade polysaccharides in the gut and produce antibodies (IgM) against the carcinoma-associated Thomsen–Friedenreich (TFα) antigen, in a dose- and time-dependent manner [58].

Several primary and branched alcohols were found to be present in both the cancer and control groups, in varying concentrations. They could be produced via the microbial fermentation of alcohols, including from methanol to heptanol [59]. Bacteria, such as Pseudomonadota (formerly Proteobacteria), generated alcohols, including *Ruminococcus*, *Prevotella*, and *Bifidobacterium,* among others, that could convert alcohols into aldehydes and SCFAs [60]. It is important to note that the alcohols in exhaled breath could be attributed to confounders, such as alcohol consumption. Furthermore, Phenol, 2-methyl-4-(1,1,3,3-tetramethylbutyl)- found in the control groups was linked to *E. aldensis*, a strain of Lachnospiraceae, which was metabolically less active and could be responsible for producing certain alcohols and SCFAs. However, the relation of Phenol, 2-methyl-4-(1,1,3,3-tetramethylbutyl)- to *E. aldensis* had not been investigated previously. We also found statistically significant correlations between the species *Candidatus Schneewindia gallinarum* and *Pseudoflavonifractor SGB15156,* and 4-ethylbenzamide (a benzamide derivative), in the healthy controls. This metabolite has been detected in the breath of smokers and emphysema patients, and has often been regarded as a possible inhibitor of *Mycobacterium tuberculosis* [61]. In sharp contrast with these findings, the association between 4-ethylbenzamide and the aforementioned bacterial taxa was not as clear. Similarly, 3-methylcyclopentyl acetate, a methyl ester resulting from the condensation of acetic acid and methanol, has been associated with Crohn’s disease and ulcerative colitis in the literature [62]. In the current study, 3-methylcyclopentyl acetate was correlated significantly to 11 of the 17 selected bacterial taxa. However, these correlations did not necessarily indicate that these bacteria had synthesized this methyl ester, since it could have originated from other alternative sources, such as environmental exposure or diet. Methacrolein, an unsaturated aldehyde and a known carcinogen, was ubiquitously correlated to the majority of the bacterial species (12 out of 17). It has been associated with lung injury and acute respiratory failure [63]. In other studies, it was reported as an oxidation product of isoprene, which has received widespread attention as a breath biomarker in several diseases, including gastric cancer.

Consequently, several classes of VOCs could be identified in exhaled breath associated with the fecal microbiome and related to different metabolic pathways. Including our results of the correlated exhaled breath metabolites in future research with a larger study cohort could further our understanding of the relationship and the origin of the metabolites. In addition to the sample size, a potential limitation in the present study could have been related to the confounding chemicals present in the ambient air, as a possible source of background bias. Similarly, the expression of certain breath VOCs in relation to the correlated bacteria is not yet fully understood. The presence of outliers in the fecal bacterial communities and their respective VOCs may suggest the presence of some rare and unclassified bacterial taxa.

We did not identify any noteworthy differences in the gut microbiome composition based solely on taxonomic composition between the gastric cancer and control groups that would enable us to distinguish between the two. This lack of differentiation was not unusual, given the limitations of the sample size, the variability of the tumor types, and the clinical stages represented in this study, as well as the restraints of microbiome DNA sequencing. While this method was not entirely quantitative, the presence of DNA alone did not necessarily indicate the production of a particular metabolite. To obtain more precise information about the processes occurring in the gut, one would need to use other approaches, such as RNA sequencing and metabolomics. Alternatively, breath analysis of VOCs has the potential to provide a less expensive, more direct, and non-invasive measure of the microbiome’s status, which would be a more practical approach in clinical settings where rapid and cost-effective testing is necessary.

In line with our findings, the established link between exhaled metabolites and the fecal microbiota could provide information on the origin of the volatile compounds in the breath, indicate the state of health, and assess recent or long-term exposure to various contaminants. Additionally, this could generate new knowledge on the mechanisms and potential of breath VOCs for gastric cancer screening in exhaled breath analysis.

## 4. Materials and Methods

### 4.1. Study Participants

This study included a total of 49 individuals, aged 24–77 years, recruited at the Riga East University Hospital, Latvia Oncology Center (Riga, Latvia), who underwent the analyses. Among them, 16 were diagnosed with gastric cancer, and 33 were healthy controls. The patient group had different clinical stages of cancer, ranging from stage IA to stage IV [64]. The study was approved by the Ethics Committee of the Riga East University Hospital Support Foundation (approval No. 18A/19), and all patients provided written informed consent before participation.

The clinical data, breath, and fecal samples were collected during the hospital visit. Breath samples were collected in a dedicated room within the oncology department for the GC–MS analysis. The fecal samples from the same participants were taken for microbiome analysis.

### 4.2. Breath Sampling

Exhaled breath samples were taken randomly at room temperature, in a room free of potential sources of VOCs, such as food, kitchen waste, drugs, solvents, and disinfectants, to prevent the risk of background bias. All participants were exposed to the same test environment, temperature, and humidity conditions, without any medical intervention. Prior to the sampling, the study subjects were advised to:

1. Fast for at least 12 h;
2. Refrain from drinking coffee, tea, and soft drinks, for at least 12 h;
3. Refrain from smoking for at least two hours;
4. Avoid alcohol intake for at least 24 h;
5. Refrain from tooth-brushing, mouthwash, or flossing;
6. Avoid chewing gum and mouth fresheners for at least 12 h;
7. Refrain from using cosmetics/fragrances prior to the procedure on the day of the test;
8. Avoid excessive physical activity (gym, jogging, cycling, and intense physical work) at least two hours prior to the test.

Prior to the breath sample collection, the medical staff interviewed the study subjects using a clinical questionnaire containing questions on personal data, VOC exposure, confounding factors, and 24 h dietary recall. Breath samples were taken using an in-house custom-made breath sampler. The breath sampler consisted of a disposable mouthpiece (Intersurgical, Wokingham, UK, 1931000) installed on a disposable elbow (Intersurgical, Wokingham, UK, 2714000S), and the CO_2_ sensor cell (Masimo, Irvine, CA, USA) was connected to the other end of the elbow. The elbow was equipped with a ¼″ port, enabling the connection of industry standard 1/4″ Tenax TA sorbent tubes. Prior to the sample collection, the sampling end of the sorbent tube was inserted into the elbow, so it protruded 5–6 mm into the interior of the elbow and was fixed with a ¼″ PTFE nut. The other end of the sampling tube was connected to a 250 mL gas-tight glass syringe (Roth, Karlsruhe, Germany) using a 1/8″ Teflon tube. The subject could freely inhale/exhale through a mouthpiece without pneumatic resistance. The sampling was achieved manually by drawing a volume of 500 mL during subsequent end-tidal exhalation segments, as identified via the CO_2_ measurement. On average, 10–15 mL of breath was drawn per exhalation. Effectively, two breath samples (each 500 mL) were taken per subject. In parallel to the breath samples, one air sample of the room was collected via a manual draw of 500 mL of ambient air into a Tenax sorbent tube. Immediately after sampling, both ends of the sorbent tube were closed with brass ¼″ nuts, and the tubes were frozen at −80 °C. Samples were stored at −80 °C and transported on dry ice. An effort was made to reduce the storage time to 4 weeks. The volatile metabolites were measured by GC–MS.

### 4.3. Chemicals and Standards for GC–MS Analysis

All standard mixtures were produced using high-purity liquid substances. Reference chemicals with stated purities of 95–99.9% were purchased from Merck (Vienna, Austria) and Fluka (Buchs, Switzerland). Standards were prepared by injecting and evaporating several microliters of the liquid compound into evacuated and heated 1 L glass bulbs (Supelco, Bellefonte, PA, USA). Next, primary standards were diluted by transferring appropriate volumes from the bulb mixtures into 3–10 L Tedlar bags (SKC Inc., Eighty Four, PA, USA), that were pre-filled with purified and humidified air (Relative Humidity 100% at 34 °C). The stainless-steel industry-standard thermal desorption tubes (1/4 in. O.D., 3½ inches long), pre-packed with Tenax TA, were purchased from Markes International Limited (Bridgend, UK). Prior to each sampling, sorbent tubes were reconditioned according to the manufacturer’s recommendations. The calibration protocols were outlined elsewhere [65].

### 4.4. GC–MS Analysis of Exhaled Breath

A two-stage thermal desorption of breath VOCs was performed using a commercial thermal desorber (UNITY, Markes International Limited, Bridgend, UK) and an autosampler (TD100, Markes International Limited, Bridgend, UK). During the primary desorption, sorbent tubes were heated to 280 °C for 6 min under a flow rate of 6.0 (99.9999%) helium at 20 mL/min. VOCs desorbed during this phase were refocused into a cold trap packed with graphitized carbon-black and maintained at 5 °C. The injection of VOCs into the capillary column was performed via the rapid heating of the cold trap to 320 °C for 1.5 min in a splitless mode.

The VOC analysis relied on an Agilent 7890A/5975C GC–MS system (Agilent, Santa Clara, CA, USA). Extracted compounds were separated using an Rxi-624Sil MS column (30 m × 0.32 mm, layer thickness 1.8 μm, Restek, Bellefonte, PA, USA) operated in a constant helium flow of 1.5 mL min^−1^. The column temperature program was as follows: 40 °C for 10 min; followed by 5 °C min^−1^ and up to 150 °C; hold for 5 min; then 10 °C min^−1^ and up to 280 °C; and finally remaining at 280 °C for 5 min. The untargeted VOC analysis was performed using the mass spectrometer working in SCAN mode with the associated m/z ranging from 20 up to 250. The peak integration was based on the extracted m/z ratio chromatograms, and this approach allowed for a proper separation of the majority of the peaks of interest from their neighbors. The quadrupole, ion source, and transfer line were kept at 150 °C, 230 °C, and 280 °C, respectively.

Identification of breath VOCs was performed in two steps. Firstly, the peak spectrum was evaluated against the National Institute of Standards and Technology (NIST) mass spectral library database. Next, the NIST identification was confirmed by comparing the respective retention times with the retention times obtained for the reference standards prepared from pure compounds, as outlined previously.

### 4.5. DNA Isolation of Fecal Samples

The study utilized sample collection bottles (Eiken Chemical Co., Ltd., Tokyo, Japan) specifically designed for fecal immunochemical occult blood testing. These bottles were used to collect, transport, and store the fecal samples. After conducting a quantitative immunochemical fecal occult blood test, all the returned bottles were frozen at −80 °C and kept frozen during the transport and storage, until they were ready for further analysis for the microbiome research. The samples were processed within 60 days from the time of collection. Prior to DNA extraction, the entirety of the buffer content containing the fecal matter (~1.5 mL) was collected from the sample bottles using a sterile, disposable syringe and then concentrated through lyophilization. The resulting lyophilized sample was used to extract microbial DNA using the MagPure stool DNA LQ (MGI Tech, Shenzhen, China) and FastPrep 24 5G instrument. Additionally, DNA was extracted from a negative control.

### 4.6. Shotgun Metagenomic Sequencing and Data Handling

MGI Tech Latvia laboratories were responsible for the sequencing process. The extracted DNA was fragmented into roughly 400 bp fragments, and a sequencing library was created using the MGIEasy PCR-Free DNA (MGI Tech, Shenzhen, China) library preparation protocol, in accordance with the manufacturer’s standard procedure. The DNBSEQ-T7 platform (MGI Tech, Shenzhen, China) was used to perform shotgun metagenomic sequencing with 150 bp paired-end reads.

The primary processing of the raw metagenomics sequencing data was conducted on high-performance computing clusters at Riga Technical University (Riga, Latvia) using an in-house Snakemake-based workflow. Adapter removal and base quality trimming was conducted with fastp tool. The host reads were extracted with Kraken2 (version 2.1.2) using the GRCh38 human reference genome. The taxonomic assignment was carried out using the MetaPhlAn4 (version 4.0.3) and vJan21_CHOCOPhlAnSGB_202103 marker database, along with Kraken2, against a database built from all available bacteria full genomes in RefSeq (accessed July 2022). A confidence value of 0.2 of the Kraken2 results was re-estimated at species level with Bracken (version 2.8) in order to improve the taxonomic abundances. The functional annotation was performed using HUMAnN 3 (version 3.6) with a full UniRef90 database (accessed December 2022), and the results were normalized at all levels by community and expressed in copies-per-million.

The results were summarized and further transformed by in-house python scripts and analyzed in an R software environment (version 4.0.5.).

### 4.7. Statistical Analysis

The statistical analysis to evaluate the connection between the VOCs from exhaled breath and the bacteria species from the fecal microbiota was carried out using CCA. The CCA found the relationship between the two multivariate sets of variables that characterized the same study participants by creating linear combinations of variables from each set (in this study, one set of combinations described the breath VOCs, and the other, the microbiome composition), so they would have a maximum correlation between them [66,67]. The CCA score plot was used to visualize the relationships (correlations) between the VOCs found in the breath and the composition of the fecal microbiota.

The most significant breath VOCs and fecal microbiome species were identified using sensitivity analyses by backward elimination of the VOCs and bacteria, and then evaluating the changes in significance of correlation among the canonical components. The least significant VOCs and bacterial species had little impact on the correlation when removed. The final set of breath VOCs and bacteria species included only the significant markers, whose removal would lead to a significant drop in correlational significance.

Since the counts of unique VOCs and unique bacterial species were significantly larger than the number of study participants, the dimensionality was reduced using S-PCA [68].

The analysis and visualization were conducted using RStudio 2022.02.3 (Build 492) and the R libraries CCA, CCP, and sparsepca. A *p*-value < 0.05 was deemed to indicate statistical significance.

## 5. Conclusions

Overall, this study investigated the significant and informative correlations between breath volatile metabolites and fecal microbiota compositions. The production of microbe-associated breath VOCs could affect the disease pathology. Using GC–MS for the VOC measurements served to complement the fecal microbiome analysis using metagenomic sequencing, in addition to an integrated approach involving a multivariate statistical analysis and bioinformatics. The proposed integrated analyses could be beneficial for a better understanding of the underlying biological processes of microbiota composition and breath VOCs. Moreover, this prospective study offers potential directions for advanced screening and developing microbiome-directed novel therapeutic targets and biomarkers for gastric cancer. Our findings further highlighted how breath VOCs were an excellent medium for the investigation of microbial metabolic functions and promising biomarker candidates that could eventually deliver on the promise of non-invasive cancer screening.

## Figures and Tables

**Table 1:** Demographic data of the study cohort.

		Cancer	Control	Total
Gender	Female	10 (62.5%)	24 (72.7%)	34 (69.4%)
	Male	6 (37.5%)	9 (27.3%)	15 (30.6%)
Age		62.2 ± 11.5	51.4 ± 14.3	54.9 ± 14.3
Stage	IA	5 (31.2%)	-	-
	IB	0 (0.0%)	-	-
	IIA	0 (0.0%)	-	-
	IIB	4 (25.0%)	-	-
	IIIA	0 (0.0%)	-	-
	IIIB	1 (6.3%)	-	-
	IIIC	0 (0.0%)	-	-
	IV	4 (25.0%)	-	-
	N/A ^1^	2 (12.5%)	-	-

^1^ N/A = not available.

**Table 2:** Descriptive analysis of the VOC expression/level between cancer and control groups (*p*-value < 0.05 is considered significant).

VOCs	All	Cancer	Control	*p*-Value (Cancer vs. Control)
	Mean ± Standard Deviation	Mean ± Standard Deviation	Mean ± Standard Deviation	
1-Decanol	374,885.551 ± 658,847.43	353,430.063 ± 615,367.78	385,288.212 ± 687,948.44	0.848
1-Dodecene	230,929.612 ± 551,407.085	123,288.563 ± 360,970.491	283,119.212 ± 621,606.394	0.356
1-Octanol	986,497.776 ± 1.123 × 10^6^	530,659.625 ± 525,202.942	1.208 × 10^6^ ± 1.269 × 10^6^	0.033
2-Pentanone	1.225 × 10^6^ ± 876,447.236	911,821.563 ± 617,815.055	1.377 × 10^6^ ± 948,989.101	0.078
2-Propanol, 1-(2-methoxy-1-methylethoxy)-	208,490.98 ± 522,305.902	181,941.5 ± 354,782.719	221,363.455 ± 591,337.416	0.701
3-Methylcyclopentyl acetate	853,658.306 ± 1.142 × 10^6^	665,181.875 ± 581,937.207	945,040.818 ± 1.331 × 10^6^	0.923
4-Ethylbenzamide	816,527.918 ± 1.398 × 10^6^	621,236.125 ± 1.211 × 10^6^	911,214.848 ± 1.488 × 10^6^	0.565
Benzene	2.426 × 10^6^ ± 4.889 × 10^6^	2.240 × 10^6^ ± 3.783 × 10^6^	2.516 × 10^6^ ± 5.397 × 10^6^	0.677
Benzene, 1-methyl-3-(1-methylethyl)-	422,211.327 ± 1.205 × 10^6^	138,661.938 ± 390,489.767	559,689.818 ± 1.430 × 10^6^	0.237
Benzyl alcohol	1.775 × 10^6^ ± 3.207 × 10^6^	1.700 × 10^6^ ± 2.987 × 10^6^	1.811 × 10^6^ ± 3.352 × 10^6^	0.662
Decane	1.089 × 10^6^ ± 1.918 × 10^6^	1.155 × 10^6^ ± 2.254 × 10^6^	1.057 × 10^6^ ± 1.770 × 10^6^	0.658
Dimethyl disulfide	105,279.388 ± 257,353.642	102,375.75 ± 143,024.5	106,687.212 ± 299,585.267	0.342
Dodecane, 5,8-diethyl-	630,501.918 ± 1.054 × 10^6^	423,613.313 ± 912,549.372	730,811.545 ± 1.115 × 10^6^	0.321
Ethanol, 2-phenoxy-	1.712 × 10^6^ ± 3.064 × 10^6^	2.214 × 10^6^ ± 4.897 × 10^6^	1.469 × 10^6^ ± 1.628 × 10^6^	0.654
Methacrolein	239,398.347 ± 420,052.987	204,225.125 ± 271,535.237	256,452.03 ± 478,734.464	0.862
Nonane	1.420 × 10^6^ ± 1.805 × 10^6^	1.203 × 10^6^ ± 1.168 × 10^6^	1.525 × 10^6^ ± 2.052 × 10^6^	0.509
Octane, 1,1′-oxybis-	1.957 × 10^6^ ± 6.918 × 10^6^	121,429.375 ± 337,233.497	2.848 × 10^6^ ± 8.320 × 10^6^	0.027
Phenol, 2-methyl-4-(1,1,3,3-tetramethylbutyl)-	3.848 × 10^6^ ± 3.645 × 10^6^	3.574 × 10^6^ ± 2.803 × 10^6^	3.980 × 10^6^ ± 4.024 × 10^6^	0.966
Toluene	4.418 × 10^6^ ± 7.580 × 10^6^	3.467 × 10^6^ ± 6.208 × 10^6^	4.879 × 10^6^ ± 8.213 × 10^6^	0.071
dl-Erythro-1-phenyl-1,2-propanediol	120,533.224 ± 325,399.861	124,073.063 ± 301,212.871	118,816.939 ± 341,011.718	0.661
n-Hexane	286,432.816 ± 842,786.914	105,393.813 ± 225,226.503	374,209.303 ± 1.009 × 10^6^	0.233

## Data Availability

The data presented in this study are available upon request from the corresponding authors.

## Supplementary Materials

The following supporting information can be downloaded at: https://www.mdpi.com/article/10.3390/molecules28083488/s1, Table S1: Correlation coefficients (breath VOCs vs. fecal bacterial taxa) for gastric cancer patients, Table S2: Correlation coefficients (breath VOCs vs. fecal bacterial taxa) for healthy controls.
